# An eHealth Application of Self-Reported Sports-Related Injuries and Illnesses in Paralympic Sport: Pilot Feasibility and Usability Study

**DOI:** 10.2196/humanfactors.8117

**Published:** 2017-11-29

**Authors:** Kristina Fagher, Jenny Jacobsson, Örjan Dahlström, Toomas Timpka, Jan Lexell

**Affiliations:** ^1^ Rehabilitation Medicine Research Group Department of Health Sciences Lund University Lund Sweden; ^2^ Athletics Research Center Department of Medical and Health Sciences Linköping University Linköping Sweden; ^3^ Department of Neurology and Rehabilitation Medicine Skåne University Hospital Lund Sweden; ^4^ Department of Health Science Luleå University of Technology Luleå Sweden

**Keywords:** epidemiology, feasibility studies, sports medicine, sports for persons with disabilities, telemedicine

## Abstract

**Background:**

Sport participation is associated with a risk of sports-related injuries and illnesses, and Paralympic athletes’ additional medical issues can be a challenge to health care providers and medical staff. However, few prospective studies have assessed sports-related injuries and illnesses in Paralympic sport (SRIIPS) over time. Advances in mobile phone technology and networking systems offer novel opportunities to develop innovative eHealth applications for collection of athletes’ self-reports. Using eHealth applications for collection of self-reported SRIIPS is an unexplored area, and before initiation of full-scale research of SRIIPS, the feasibility and usability of such an approach needs to be ascertained.

**Objective:**

The aim of this study was to perform a 4-week pilot study and (1) evaluate the monitoring feasibility and system usability of a novel eHealth application for self-reported SRIIPS and (2) report preliminary data on SRIIPS.

**Methods:**

An eHealth application for routine collection of data from athletes was developed and adapted to Paralympic athletes. A 4-week pilot study was performed where Paralympic athletes (n=28) were asked to weekly self-report sport exposure, training load, general well-being, pain, sleep, anxiety, and possible SRIIPS. The data collection was followed by a poststudy use assessment survey. Quantitative data related to the system use (eg, completed self-reports, missing responses, and errors) were analyzed using descriptive statistics. The qualitative feasibility and usability data provided by the athletes were condensed and categorized using thematic analysis methods.

**Results:**

The weekly response rate was 95%. The athletes were of the opinion that the eHealth application was usable and feasible but stated that it was not fully adapted to Paralympic athletes and their impairments. For example, it was difficult to understand how a new injury or illness should be identified when the impairment was involved. More survey items related to the impairments were requested, as the athletes perceived that injuries and illnesses often occurred because of the impairment. Options for description of multifactorial incidents including an injury, an illness, and the impairment were also insufficient. Few technical issues were encountered, but athletes with visual impairment reported usability difficulties with the speech synthesizer. An incidence rate of 1.8 injuries and 1.7 illnesses per 100 hours of athlete exposure were recorded. The weekly pain prevalence was 56% and the impairment contributed to 20% of the reported incidents.

**Conclusions:**

The novel eHealth-based application for self-reported SRIIPS developed and tested in this pilot study was generally feasible and usable. With some adaptation to accommodate Paralympic athletes’ prerequisites and improved technical support for athletes with visual impairment, this application can be recommended for use in prospective studies of SRIIPS.

**Trial Registration:**

ClinicalTrials.gov NCT02788500; https://clinicaltrials.gov/ct2/show/NCT02788500 (Archived by WebCite at http://www.webcitation.org/6v56OqTeP)

## Introduction

Paralympic sport continues to grow and attracts athletes from all around the world. However, participation in Paralympic sport is, like all sport, associated with a risk of sports-related injuries and illnesses, and Paralympic athletes’ additional medical issues are challenging to health care providers and medical staff [1].

Knowledge of sports-related injuries and illnesses in Paralympic sport (SRIIPS) is limited, and few prospective studies have assessed SRIIPS over time [2-4]. During the Paralympic Games in London 2012 and Sochi 2014, considerably higher injury incidences were recorded compared to the corresponding Olympic Games [5,6]. Paralympic athletes also have higher illness incidence rates compared to Olympic athletes [7]. To improve health and safety in Paralympic sport, there is a need for prospective longitudinal monitoring of SRIIPS over entire training seasons to determine distributions and etiological mechanisms [8,9]. To advance knowledge of the incidence and risk factors of SRIIPS, we have initiated a prospective longitudinal study using eHealth-based data collection of self-reports [10].

To allow data collection over longer periods of time and in heteregenous populations, athlete monitoring through self-reports is an established method of observing athletes’ health, including both sports-related injuries and illnesses [11-13]. Self-reports enable collection of information on overall health based on simultaneous recording of injuries, physical and mental illnesses, sports exposure, training load, and risk factors, specifically adapted to the sports population of interest [8,14,15]. Moreover, self-reports provide more realistic data than reports by medical personnel who may underestimate the injury rates compared to athletes themselves [16].

By collecting data electronically, self-reports can be used with minimal memory bias and constitute real-time personalized data [17]. Advances in mobile phone technology and networking systems offer novel opportunities to develop innovative eHealth applications to collect data [18]. However, most studies have only included able-bodied athletes, and studies using eHealth applications in Paralympic athletes with various physical, intellectual, and visual impairments are lacking.

For successful implementation of an application, it is important to consider methodological and practical challenges [19,20]. Pilot studies allow the development and testing of the method and give advance warnings about where the forthcoming main research project could fail [21]. Potential sources of errors could be poor definitions, difficulties in interpreting questions and data, and failure to use the system. Establishing a user-friendly surveillance system that targets the population is therefore a key factor [8,22]. Thus, before initiation of full-scale research, a pilot study focusing on feasibility and usability issues is needed to ascertain the ability to use the new application for future data collection [23]. As Paralympic sport includes athletes with a wide range of impairments [1], the eHealth application must allow adaptation to users’ specific needs and circumstances [24]. This is to ensure that they will be able to adopt the new monitoring system in daily procedures, regardless of their impairments, and that the output is experienced as useful for them [8,22].

The aim of this study was to perform a 4-week pilot study and (1) evaluate the monitoring feasibility and system usability of a novel eHealth application for longitudinal epidemiological research on self-reported SRIIPS and (2) report preliminary data on SRIIPS.

## Methods

### Development of the eHealth Application

The purpose of the eHealth monitoring is to enable Paralympic athletes to self-report SRIIPS, exposure to sport, and general health parameters in an e-diary. For the data collection, the Briteback survey tool was used. This tool is integrated with software built on team communication research. The tool allows researchers to construct specific surveys, which are sent automatically as Web links in emails and text messages. The surveys are adapted to computers, tablets, and mobile phones, and participants can choose how to enter their data. Automated system-generated statistics are provided immediately after reporting of data.

The prototype eHealth application was developed and adapted to Paralympic athletes based on a theoretical foundation of existing research within sports medicine [12,13,25], Paralympic athletes’ own perceptions of experiences of sports-related injuries [26], our study protocol [10], and the Web Content Accessibility Guidelines 2.0 (WCAG 2.0) [27]. The main focus was to include features that are specific to Paralympic athletes. For example, pain, involvement of the impairment, and already existing medical issues may be present [26]. The research team, consisting of sports injury epidemiologists, physicians, physical therapists, and disability researchers together with computer scientists and athletes adapted and tested the system for Paralympic athletes.

**Figure 1:**
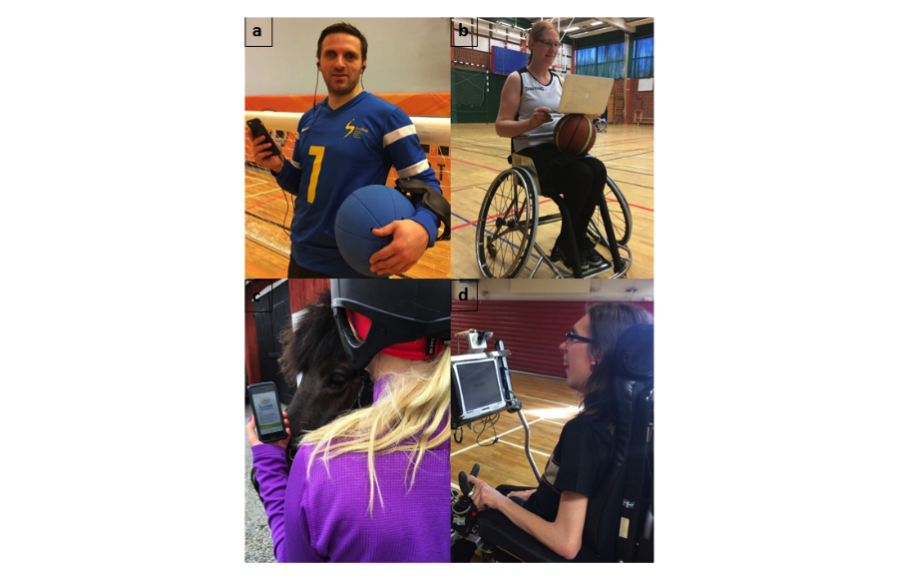
Survey design and technology formulated for use among able-bodied athletes need adaptations to Paralympic athletes with a broad range of impairments. (A) Visually impaired athlete using speech synthesizing technology adapted to the eHealth application, (B) Wheelchair basketball player with individual training behavior often without coach and medical staff, (C) Athlete often traveling using the eHealth application in her training environment, (D) Athlete with cerebral palsy and tetraplegia using a joystick to navigate the eHealth application.

To evaluate a Web tool as feasible and usable for users with disabilities, the WCAG 2.0 guidelines require it to be perceivable, operable, understandable, and robust for all categories of users [27]. Therefore, a central requirement of the eHealth application was that athletes with a visual impairment, physical impairment, or intellectual impairment (Figure 1) could use it at the same conditions. To make the content usable to the athletes, the eHealth application was developed to meet the WCAG 2.0 accessibility guidelines. Principles related to user interface design, screen resolution, keyboard navigation, avoidance of seizure-causing content, and avoidance of content that causes mistakes were considered in the development. The application should also appear and operate in predictable ways, and the users should have enough time to read and use the content [27].

The final weekly e-diary consisted of 12 questions for athletes to respond to pertaining to the following topics:

- Participation in normal training
- Exposure to sport (sessions)
- Exposure to sport (hours)
- Exposure to competition
- Rate of perceived exertion
- Use of analgesics
- General well-being
- Sleep
- Anxiety
- Pain
- New injury
- New illness

Depending on responses, subquestions related to reported SRIIPS could also appear.

### Study Population

A pilot study cohort stratified to represent the different impairments, genders, and sports was selected in June 2016 from the Swedish Paralympic Program. The following inclusion criteria, adopted from the study protocol [10], were used: age 18 to 55 years; being a registered athlete within the Swedish Paralympic Program; being classified as an eligible International Paralympic Committee athlete with visual impairment, physical impairment, or intellectual impairment; being able to communicate in Swedish; and having the opportunity to answer an e-diary weekly during 4 weeks. A total of 37 elite athletes were invited to participate, and 28, 9 women and 19 men (aged 20 to 51 years) with visual impairment (n=11), physical impairment (n=15), and intellectual impairment (n=2), accepted the invitation. The athletes were active in the following para-sports: shooting, canoeing, goalball, athletics, judo, swimming, boccia, cycling, table tennis, wheelchair rugby, cross-country skiing, wheelchair curling, and ice hockey. Four athletes, all with physical impairment, declined participation because of lack of time prior to the Paralympic Games 2016. Five athletes never responded, 3 with physical impairment and 2 with intellectual impairment.

### Ethical Considerations

The study followed the ethical principles for medical research involving human subjects per the World Medical Association Declaration of Helsinki and the Strengthening the Reporting of Observational Studies in Epidemiology (STROBE) guidelines and is registered at ClinicalTrials.gov [NCT02788500]. The entire study was approved by the Regional Ethical Review Board in Lund, Sweden (Dnr 2016/169). Participation in the study was voluntary, and informed written consent was collected from all participants.

### Feasibility and Usability: Theoretical Framework

Feasibility studies enable researchers to assess if a study design and preliminary results can be shaped into relevant findings and future interventions. It is necessary to pursue a feasibility study if (1) there are few previously published studies in the research area, (2) a specific intervention is used, and (3) the study population requires unique consideration of the method.

Feasibility can be referred to as the ability of users to adopt a new system in daily procedures with focus on the questions: Can it work? Does it work? and Will it work? Important aspects of feasibility in this study were acceptability (Is the application suitable?), demand (Is the application likely to be used?), practicality (Can the application be used outside the intervention?), adaptation (Will the application work for this population?), integration (Can the application be integrated in an existing system?), expansion (Can the application be expanded?), and implementation (Can the application be successfully delivered to the participants?) [19].

Usability is a characteristic of quality in use, according to the International Organization for Standardization [28]. It denotes whether a system can be used technically by specified users to achieve goals with regard to (1) learnability (how easy users can learn the system), (2) efficiency (being able to complete a task), (3) effectiveness (the amount of effort required to complete a task), (4) satisfaction (the degree to which the user was happy with the experience while performing a task), and (5) error recovery (the users should make few errors, and errors should be easy to recover from) [28,29]. An important context of usability in this project was to ensure that an athlete with the expected ability due to their impairment can use the system and that the application is technically available to all potential users [30].

The Fit between Individuals, Task, and Technology (FITT) framework of information technology (IT) adoption was used to structure and present the data on feasibility and usability goals (Table 1). FITT suggests that IT adoption in health care is dependent on socio-organizational-technical factors including task-technology fit, individual-task fit, and individual- technology fit [31].

**Table 1:** Feasibility and usability goals structured according to the Fit between Individuals, Task, and Technology framework and the Post-Study System Usability Questionnaire.

Conceptual framework and measure			Data source
**Feasibility**			
	**Individual**		
		Demographics (gender, age, sport, impairment)	Athlete information
		Fit to individual	PSSUQ^a^ Data from the eHealth application (ie, missing answers, impairment related problems)
	**Task**		
		Fit into daily routines	PSSUQ Data from the eHealth application (ie, answer frequency)
		Fit into Paralympic sport	PSSUQ Data from the eHealth application (ie, number of reported incidents, type of reported incidents). Interest from athletes and organization
**Usability**			
	**Technology**		
		Efficiency	PSSUQ Data from the eHealth application (ie, athlete workflow)
		Effectiveness	PSSUQ
		Learnability	PSSUQ
		Satisfaction	PSSUQ
		Error recovery	Reported and detected errors

^a^PSSUQ: Post-Study System Usability Questionnaire.

**Definitions of an injury and an illness.**

Injury:

Any new musculoskeletal pain, feeling, or injury that causes changes in normal training or competition to the mode, duration, intensity, or frequency, regardless of whether or not time is lost from training or competition

Illness:

Any new illness or psychological complaint that causes changes in normal training or competition to the mode, duration, intensity, or frequency, regardless of whether or not time is lost from training or competition

For example, IT adoption in an athletic environment may depend on the fit between the attributes of the individual user (ie, motivation, experience, computer anxiety), attributes of the technology (ie, functionality, usability), and attributes of the task (ie, complexity, task, organization).

### Data Collection

A 4-week SRIIPS pilot study was performed with an integrated poststudy feasibility and usability assessment [18,24]. The athletes were asked to weekly report sport exposure, training load, general well-being, pain, sleep, anxiety, and possible SRIIPS, according to the definitions in the SRIIPS study protocol (Textbox 1) [10]. The first author (KF) followed up on all data and any technical issues every week. After having completed the 4-week pilot study, the athletes were asked to assess the method using open questions related to the feasibility and usability (Table 1) [19,29] and a modified version of the Post-Study System Usability Questionnaire (PSSUQ) [32]. This is a questionnaire that was developed to assess user satisfaction after participation in scenario-based usability studies. With the PSSUQ, the researchers can understand which aspects of the computer system the users are particularly concerned with and which aspects they are satisfied with [32].

### Data Analysis

Quantitative data related to demographics, system use, completed self-reports, number of reported incidents, missing answers, and system errors were analyzed using descriptive statistical methods.

The qualitative feasibility and usability data were condensed and categorized using a thematic analysis method. Thematic analysis is a flexible method for identifying, analyzing, and reporting patterns within various data sets (eg, texts, webpages, and interviews). The method provides rich and detailed information that is associated with the specific research question [33]. The focus here was on identifying opinions about the eHealth application, detecting methodological issues, and determining if the method matched the users’ needs and behavior. Sentences containing aspects of relevance to feasibility and usability were transformed to themes, codes, and meaning units.

Data on SRIIPS collected during the 4-week period were analyzed using basic descriptive statistics. The incidence rates were calculated as the number of new incidents divided by total athlete exposure hours (per 1000 hours of sport participation) [10].

## Results

### Quantitative Poststudy Feasibility and Usability Evaluation

A total of 1643 self-reports, 1354 weekly e-diary reports, and 289 responses to follow-up questions were collected. The average weekly response rate was 95%. A total of 37 instances of missing data were noted in the weekly e-diary reports; 28 were observed among athletes with visual impairment, 7 from athletes with physical impairment, and 2 from athletes with intellectual impairment. Questions concerning pain, anxiety, and training load generally had a high response rate (96% to 100%). The questions with most missing answers (n=11) were about general well-being with horizontally displayed check boxes. The follow-up questions, for example, concerning SRIIPS symptoms, diagnosis, and injury severity, had on average 1 to 2 missing answers every week; 11 of these were from athletes with visual impairment and 2 from athletes with physical impairment. A total of 21 athletes, 8 with visual impairment, 12 with physical impairment, and 1 with intellectual impairment, provided complete postuse feasibility and usability data. Two technical errors related to the system and the speech synthesizer were reported by athletes with visual impairment. No system use errors occurred. Almost three-quarters (15/21, 71%) of the athletes reported that it was easy to complete the task. About three-quarters (16/21, 76%) of the athletes found it easy to define a new illness, and 52% (11/21) found it easy to define a new injury. About three-quarters (15/21, 76%) of the athletes reported that it was easy to use the closure form, and 62% (13/21) reported that the application was adapted to Paralympic sport. Most (18/21, 86%) of the athletes were satisfied with the experience of performing the task, and 90% (19/21) found it important to perform this study.

### Qualitative Poststudy Feasibility and Usability Evaluation

A summary of the thematic analysis is presented in Table 2.

#### Health Monitoring in Paralympic Sport

The athletes’ opinion was that some parts of the eHealth application were not fully adapted to Paralympic athletes. For example, the athletes found it difficult to know how to define and identify a new injury or illness, especially when their impairment was involved. In addition, more survey items related to an impairment were requested, as the perception was that some incidents occurred because of the impairments. The athletes also found it important to be able to report all new injuries and illnesses (ie, also injuries that had not been sustained during sports participation).

**Table 2:** Summary of the thematic analysis of the Paralympic athletes’ feasibility and usability evaluation of the eHealth application.

Theme	Code	Meaning unit
Health monitoring in Paralympic sport	Feasibility to Paralympic athletes	The application is not specifically adapted to Paralympic sport
		It is difficult to define a new SRIIPS^a^
		Some injuries occur because of the impairment
Survey design	Complex incidents	It is difficult to report several injuries or illnesses
		Insufficient description of multifactorial incidents
		More free text alternatives and multiple check box alternatives would be good
Impairment diversity and usability	Usability to visually impaired athletes	It is not trouble-free to use a screen reader
		Horizontal questions do not work with VoiceOver
		It is easier to use free text alternatives
Longitudinal eHealth monitoring	Sustainability	It is easy to understand and follow the weekly e-diary
		The terminology used is intelligible
		It is important that this kind of study is conducted

^a^SRIIPS: sports-related injuries and illnesses in Paralympic sport.

#### Survey Design

Identified issues were also related to the survey design and were associated with the reporting of complex incidents using the survey design originally developed for able-bodied athletes. For example, if an athlete wanted to report 2 new injuries in the weekly report, they did not easily understand how to accomplish this task.

The perception was also that there were insufficient options for describing multifactorial incidents including an injury, an illness, and the impairment. To improve the design, the athletes asked for opportunities to better describe their incidents through free text or more multiple check box alternatives.

#### Impairment Diversity and Usability

Athletes with visual impairment had usability difficulties with tasks involving a visual analog scale and horizontal reply alternatives due to a technical problem with the connection between their speech synthesizer and the eHealth application. Some athletes with visual impairment chose instead to write free text at the end of the questionnaire or not leave a response at all. The questions using vertically displayed response alternatives worked well for the athletes with visual impairment. Athletes with physical impairment or intellectual impairment did not report any functionality problems.

#### Longitudinal eHealth Monitoring

The athletes stated that the use of the eHealth application was feasible and could be extended to longer periods of time. They perceived that it was easy to understand and use the application. Most of the athletes were of the opinion that the terminology was comprehensible and that it was easy to understand which dates and week they should report. A majority also stated that it is important that health monitoring is performed.

### Data on Sports-Related Injuries and Illnesses in Paralympic Sport

One athlete dropped out during the study period; thus, 4-week data were available from 27 athletes. A total of 10 athletes (37%) reported anxiety, 15 (56%) reported pain, and 9 (33%) reported use of analgesics weekly. The median self-rated general well-being score was 4 (1-7). The average time spent on training each week was 7.6 hours. The median weekly rated perceived exertion was 6 (1-10). In total, 15 new injuries (reported by 12 athletes) and 14 new illnesses (reported by 12 athletes) were reported, giving an incidence rate of 1.8 injuries per 100 hours and 1.7 illnesses per 100 hours of athlete exposure, respectively. For 71% (5/7) of the injuries and 60% (6/10) of the illnesses, the athlete reported a higher mean training load than the week before. Tissue inflammation and pain (10/15, 67%) and upper respiratory tract infections (9/14, 64%) were the most common preliminary causes. A total of 80% (12/15) of the injuries were related to overuse, 66% (10/15) of the injuries were reported from athletes with visual impairment, and 57% (8/14) of illnesses were reported from wheelchair athletes. The typical injury severity was 1 to 3 days of time loss of training and 2.6 missed training sessions for illnesses. In 20% (3/15) of the injuries and 21% (3/14) of the illnesses, the impairment was perceived to be involved in the cause.

## Discussion

### Principal Findings

Advances in eHealth technology for athlete self-reporting and monitoring [34] have been rapid; however, the sport-specific functionality and usefulness of surveillance measures have rarely been established. Data with poor quality may thereby in the end cause problems with developing preventive measures [22]. Therefore, considering design quality and the meaning of data along with effective utilization of technology is crucial in the implementation of self-report measures [11]. Especially smaller feasibility studies with mixed methods have been shown to yield innovative results [19]. This led us to develop and test the eHealth application of self-reported SRIIPS specifically adapted to Paralympic athletes in this pilot study with particular focus on feasibility and usability. In summary, we found eHealth-based monitoring of self-reports of Paralympic athletes’ health to be generally feasible and usable with regard to fitting into daily routines and using technology. However, the study revealed some critical factors, mostly related to the fit to Paralympic sport, which should be accommodated before this application can be used in full-scale research. It is also recommended that these critical factors be considered in existing and future injury and illness surveillance systems.

### Feasibility and Usability

A critical conceptual issue related to feasibility and the fit between the individual, task, and technology was how to define and report new SRIIPS, especially when the impairment was involved. The athletes perceived that the eHealth application was not fully adapted, as some SRIIPS may occur because of the impairment. This observation corroborates the reports from a recent qualitative study where Paralympic athletes perceived that their impairments played an important role in the etiology of SRIIPS [26]. Moreover, a high prevalence of pain may complicate the process of defining and distinguishing a new sports injury from existing pain related to the impairment. This emphasizes the importance of adaptations of surveillance systems to the specific sport population, here Paralympic athletes’ various and complex impairments. Thus, the use of questionnaires developed for able-bodied athletes cannot directly be transferred to Paralympic athletes without specific adaptations, such as, for example, visual impairments [35].

Regarding usability efficiency, the athletes described that there were not enough options for description of multifactorial incidents including injuries, illnesses, and impairments. The construction of questions and terminology has previously been reported to be a main issue identified by athletes, and athletes are more willing to complete surveillance systems if they can recognize themselves in the questions asked [20]. Accordingly, the survey design has been further developed following this pilot study. The definition of SRIIPS has been clarified, the survey items better adapted to Paralympic sport, additional alternatives related to the impairment have been added, the possibilities to report multifactorial incidents extended, and more examples and free text alternatives provided to improve athlete satisfaction and motivation. One of the most important objectives in self-report measures is to collect meaningful data in relation to the needs of the athletes [11]. Thus, it is crucial that data related to the impairment are routinely collected when SRIIPS are monitored in order to ensure study feasibility and usability.

Another usability design issue related to task completion was the human-computer error of the audible feedback system used by the athletes with visual impairment. Even though there have been developments of touch screen devices, many are still inaccessible to visually impaired users who often adopt error recovery compensatory strategies [36]. Electronic questionnaires that are too difficult to use may discourage responses and reduce data quality [37]. Some of the parameters (eg, the visual analog scale and horizontal Likert scales) will be slightly modified for athletes with visual impairment. The system worked well for athletes with physical impairment and athletes with intellectual impairment without any major learnability or error recovery issues. The relative lack of technical problems and barriers encountered is not surprising as the application met most of the accessibility criteria recommended in WCAG 2.0 and was adapted to Paralympic athletes’ own perceptions of experiences of sports-related injuries [26,27].

### Monitoring Sustainability

Possible explanations for the high response rate are the short study period and system usability adaptation for easy use on mobile phones and other platforms. A restriction in athlete monitoring using self-reports is the workload assigned to the athlete, implying that collection of as little and as relevant data as possible is important in long-term surveillance [11].

The athletes were of the opinion that the application was easy to understand and could be extended to longer periods of time. Thus, we considered the application to be feasible for Paralympic athletes and believe that it can be adopted in their daily procedures with regard to the ability of the users [38]. Finch et al [34] recently described that, along with the development of digital tools, data can favorably be collected in real time from athletes and not by the medical teams, which has also proven feasible in other studies [12,13].

### Data on Sports-Related Injuries and Illnesses in Paralympic Sport

Only 2 similar studies within Paralympic sport have included athlete exposure based on time [39,40]. For effective implementation of prevention strategies, incidence based on athlete exposure is a key factor [41]. A limitation of these 2 studies [39,40] is that the inclusion of injuries only referred to trauma and medical attention. In our study, 80% of the reported injuries were related to overuse, which indicates the importance of using an injury definition in Paralympic sport that also includes these types of injuries. In addition, the observed high prevalence of pain and relatively high use of analgesics raises concerns about Paralympic athletes’ health. Few studies have assessed the prevalence, causes, and behaviors associated with pain among Paralympic athletes, and further research on this topic is warranted.

Only a handful of studies have assessed the incidence of illnesses among Paralympic athletes. Studies at the Paralympics Games indicate that illness rates are similar to injury rates [25]. This was also found in our study as well. It is therefore important that illnesses are included in athlete monitoring, well in line with the recommendations of future research priorities [34].

### Strengths and Limitations

A strength of this study is the detailed preparatory work undertaken to develop the eHealth application and specifically adapt it to Paralympic athletes with visual impairment, physical impairment, and intellectual impairment. Another strength is the subsequent evaluation and correction of feasibility and usability indicators of the monitoring system before the start of full-scale long-term studies. A limitation is that we only evaluated poststudy reported feasibility and usability issues and that the qualitative analysis included only written answers and no interviews. Another limitation is that the pilot study period was relatively short, and it is therefore not possible to distinguish long-term results and response rates. A larger study sample including athletes from all Paralympic sports may also have provided further insights into the feasibility and usability of this novel eHealth application.

### Conclusion

The novel eHealth-based application for self-reported SRIIPS developed and tested in this pilot study was generally feasible and usable. With some adaptation to accommodate Paralympic athletes’ prerequisites and improved technical support for athletes with visual impairment, this application can be recommended for use in prospective studies of SRIIPS. This will advance our knowledge of the incidence and risk factors of SRIIPS and facilitate the development of evidence-based prevention measures adapted to Paralympic sport.

## Abbreviations

FITT: Fit between Individuals, Task, and Technology
IT: information technology
PSSUQ: Post-Study System Usability Questionnaire
SRIIPS: sports-related injuries and illnesses in Paralympic sport
STROBE: Strengthening the Reporting of Observational Studies in Epidemiology
WCAG: Web Content Accessibility Guidelines
